# NVP-BEZ235 inhibits thyroid cancer growth by p53- dependent/independent p21 upregulation

**DOI:** 10.7150/ijbs.37592

**Published:** 2020-01-14

**Authors:** Banjun Ruan, Wei Liu, Pu Chen, Rongrong Cui, Yu Li, Meiju Ji, Peng Hou, Qi Yang

**Affiliations:** 1 Department of Endocrinology, The First Affiliated Hospital of Xi'an Jiaotong University, Xi'an 710061, P.R. China; 2 Center for Translational Medicine, The First Affiliated Hospital of Xi'an Jiaotong University, Xi'an 710061, P.R. China; 3 Key Laboratory for Tumor Precision Medicine of Shaanxi Province, the First Affiliated Hospital of Xi'an Jiaotong University, Xi'an 710061, P.R. China

**Keywords:** NVP-BEZ235, Thyroid cancer, PI3K/Akt/mTOR, p53, Cell cycle arrest, p21

## Abstract

NVP-BEZ235 is a novel dual PI3K/mTOR inhibitor, currently in phase 1/2 clinical trials, exhibiting clinical efficiency in treatment of numerous malignancies including thyroid cancer. Cancer cells harboring mutant p53 was widely reported to be blunt to pharmaceutical therapies. However, whether this genotype dependent effect also presents in thyroid cancer when treated with NVP-BEZ235 remains unknown. Therefore, in this study, the tumor suppressing effects of NVP-BEZ235 in thyroid cancer cell lines and *in-vivo* xenograft mouse model harboring different p53 status were examined. The antitumor effects were confirmed in p53 mutant thyroid cancer cells, though less prominent than p53 wild type cells. And for the p53 mutant cells, p53-independent upregulation of p21 plays a critical role in their response to NVP-BEZ235. Moreover, GSK3β/β-catenin signaling inhibition was implicated in the p21-mediated G_0_/G_1_ cell cycle arrest in both p53 wild type and mutant thyroid cancer cells treated with NVP-BEZ235.

## Introduction

Thyroid cancer is the most frequent endocrine cancer, and its incidence has rapidly increased over the past few decades all over the world, comprising a broad spectrum of diseases with variable prognoses 1-3. Although the death rate of thyroid cancer is relatively low, the rate of disease recurrence or persistence is high, which is associated with increased incurability and patient morbidity and mortality 4. Thyroid cancers derive from endocrine thyroid cells, including follicular thyroid cells and parafollicular C cells, and is classified into several histological kinds according to the cellular origins, characteristics and prognoses. Papillary thyroid cancer (PTC) and follicular thyroid cancer (FTC) pertain to well-differentiated carcinomas (WDTCs), which not only could be effectively cured by surgical and radio iodinated therapy, but also acquire a favorable outcome. On the contrary, poorly differentiated thyroid cancer (PDTC) and anaplastic thyroid cancer (ATC), belong to the minority malignancies, however with extremely high mortality and lack of efficient therapeutics 5. Thyroid cancer initiation and progression occurs through gradual accumulation of various genetic and epigenetic alterations, such as activating mutations of the *BRAF^V600E^*, *RET/PTC* rearrangement, *PTEN* deficiency, and *TP53* mutations, that involve the activation of MAPK and PI3K/AKT signaling pathways 5, 6. Novel compounds and therapeutic strategies that selectively target these pathways have been identified, some of which have been evaluated in preclinical and clinical studies 5-8.

NVP-BEZ235 is an orally bioavailable imidazoquinoline derivative that inhibits the activity of PI3K/mTOR, currently in phase 1/2 clinical trials 9. Therapeutic potency of NVP-BEZ235 has achieved efficacy as a monotherapeutic drug or in combination with kinds of anticancer drugs. Particularly, NVP-BEZ235 treatments enhance sensitivity to other drugs and improve drug resistance in numerous malignancies, including acute myeloid leukemia 10, prostate cancer 11, ovarian cancer 12, non-small cell lung cancer 13, gastric cancer 14, colorectal cancer 15, and thyroid cancer 16.

Nuclear transcription factor p53 have been identified as a tumor suppressor gene attributed to its role in inducing cell cycle arrest and/or apoptosis 17. However, loss of function mutations in *TP53* gene always occur in over 50 % of human cancers and *TP53* then acts as an oncogenic gene in these malignant cancer cells 18. Tumors carrying *TP53* mutations display a chemotherapy-resistant phenotype 19. In some cases, *TP53* mutations are observed in thyroid cancer and exacerbates the malignant phenotype of the tumors 20. Though NVP-BEZ235 has been verified efficacy in treating thyroid cancer 16, its antitumor effects and mechanisms in thyroid cancer harboring *TP53* mutations remain largely unknown. In this study, we used a panel of authenticated thyroid cancer cell lines carrying wild-type *TP53* or mutant-type *TP53* to test the therapeutic potential of NVP-BEZ235 and attempted to understand its anticarcinogenic mechanisms in thyroid cancer.

## Materials and Methods

### Cell lines and cell culture

Human thyroid cancer cell lines BCPAP, IHH4, K1 were obtained from Dr. Haixia Guan (The First Affiliated Hospital of China Medical University, Shenyang, China). C643 was provided by Dr. Lei Ye (Ruijin Hospital, Shanghai, China). Among them, IHH4 and K1 cells harbor wild-type *TP53*, whereas BCPAP and C643 cells carry mutant-type *TP53*. Genetic alterations including *TP53* genomic status for each cell line were summarized in **Table 1**. All cell lines were cultured in RPMI 1640 medium supplemented with 10 % fetal bovine serum (FBS) at 37 °C in an incubator containing 5 % CO_2_.

### NVP-BEZ235 or GSK3β inhibitor treatment

NVP-BEZ235 was purchased from Cayman Chemical Co. (Ann Arbor, MI, USA), and dissolved in dimethylsulfoxide (DMSO). Cells were treated with indicated concentration of NVP-BEZ235 (0 nM, 50 nM, 100 nM, 200 nM, 500 nM and 1000 nM, respectively). The medium and agent were replenished every 24 h at the incubation period. DMSO was added to the medium as the vehicle control and the concentration was always kept below 0.1 %. In addition, SB216763, a GSK3β inhibitor, were purchased from TargetMol (Boston, MA, USA) and used at a concentration of 10 μM in this study.

### p21 or p53 knockdown

A double-stranded oligonucleotide of siRNA targeting p21 (5′-AGACCATGTGGACCTGTCA-3′) or p53 (5′-AGACCUAUGGAAACUACUU-3′), and negative control (NC) siRNA were obtained from Ribobio (Guangzhou, China). Cells were transfected at 50 % confluence using X-tremeGENE siRNA transfection reagent (Roche, Germany) with a final siRNA concentration of 50 nM.

### RNA extraction and quantitative real-time PCR (RT-qPCR) analysis

Total RNA was extracted from cells using TRIzol® Reagent (Invitrogen, Carlsbad, CA, USA) according to the manufacturer's instructions. RNA samples (1 μg) were reverse transcribed to cDNA by PrimeScriptTM RT reagent Kit (Takara, Dalian, China) according to the instructions of the manufacturer. Real-time quantitative PCR was performed on a CFX96 real-time PCR system (Bio-Rad, Hercules, CA, USA) using SYBR Premix Ex TaqTM (Takara, Dalian, China) and results were normalized to *β-actin*. Each sample was run in triplicate. Primer pairs of *CDKN1A* and *β-actin* used for amplification in this study are: 5'-GGCGGCAGACCAGCATGACAGATT-3' (*CDKN1A* forward) and 5'-GCAGGGGGCGGCCAGGGTAT-3' (*CDKN1A* reverse); 5'-GCACAGAGCCTCGCCTT-3' (*β-actin* forward) and 5'-GTTGTCGACGACGAGCG-3' (*β-actin* reverse).

### Western blot analysis

Treated cells were lysed in prechilled RIPA buffer containing protease inhibitors.

Equal amount of proteins was electrophoresed using 10 % SDS-PAGE, and transferred onto polyvinylidene fluoride (PVDF) membranes (Roche Diagnostics, Mannheim, Germany). The membranes were blocked in 5 % BSA dissolved in TBST (1 × TBS buffer and 0.05 % Tween 20) for 2 h, and then incubated at 4 ℃ overnight with the indicated primary antibodies. Anti-phospho-Akt (Ser473 or Thr308; p-Akt) and anti-total Akt (t-Akt) antibodies were purchased from Bioworld Technology (St. Louis, MO, USA). Anti-total mTOR (t-mTOR), anti-p53, anti-β-catenin, anti-phospho-β-catenin (Ser33; p-β-catenin) and anti-Cdk2 antibodies were purchased from Santa Cruz Biotechnology (Dallas, TX, USA). Anti-phospho-mTOR (Ser2448; p-mTOR), anti-FOXO3a, anti-phospho-FOXO3a (Thr32; p-FOXO3a), anti-p21, anti-GSK3β and anti-phospho-GSK3β (Ser9; p-GSK3β) antibodies were purchased from Cell Signaling Technology (Beverly, MA, USA). Anti-cyclin E1 antibodies was purchased from Sino Biological (Beijing, China). Anti-GAPDH was purchased from Abmart (Shanghai, China). Then species-specific HRP-conjugated secondary antibodies were incubated (ZSGB Biotechnology, Beijing, China) and immunoblotting signals were visualized using the Western Bright ECL detection system (Advansta, CA, USA).

### MTT assay

3-(4,5-Dimethylthiazolyl-2)-2,5-diphenyltetrazolium bromide (MTT) assay was performed to evaluate cell proliferation. Cells (3~5 × 10^3^/well) were seeded in 96-well plates and cultured with various concentrations of NVP-BEZ235. IC_50_ values were calculated using the Reed-Muench method. After treatment, 20 μL of MTT (5 mg/ml; Sigma, Saint Louis, MO, USA) was added and incubated for additional 4 h. Then the MTT containing medium was discarded, followed by addition of 150 μL of DMSO. The plates were then read on a microplate reader using a test wavelength of 570 nm and a reference wavelength of 670 nm. All the experimental sets were performed in triplicate.

### Colony formation assays

Cells were seeded onto 6-well plates at a density of 800 cells per well. After 24 h of culture, the cells were treated with NVP-BEZ235 (200 nM) or DMSO vehicle. The medium and drug was refreshed every 3 days. After 14 days of culture, surviving colonies (≥ 50 cells per colony) were stained with 0.5 % crystal violet in 20 % methanol for 20 minutes and counted. Each experiment was performed in triplicate.

### Flow cytometry analysis

For cell cycle assay, cells in the exponential growth phase were serum starved for 12 h. After co-culture with 200 nM NVP-BEZ235 for 48 h, cells were harvested, washed twice in phosphate buffered saline (PBS), and fixed in ice-cold 70 % ethanol for at least 30 min. Cells were then stained with propidium iodide (PI) solution (50 μg/ml PI, 50 μg/ml RNase A, 0.1 % Triton X-100, 0.1 mM EDTA). Cell cycle distributions were assessed based on DNA contents using a flow cytometer (BD Biosciences, Franklin Lakes, NJ, USA).

### Immunofluorescence

The human thyroid cancer cells were seeded onto glass coverslips in 6-well plates and cultured until 50-60 % confluence, followed by treatment with 200 nM NVP-BEZ235 for 48 h. Cells were then fixed with 4.0 % paraformaldehyde for 20 min at room temperature and blocked in 5.0 % goat serum for 30 min. Next, the coverslips were incubated at 4 °C with primary FOXO3a antibody (#2497, Cell Signaling Technology,) overnight. Subsequently, the cells were washed in PBS. The coverslips were incubated with goat anti-rabbit IgG H&L (FITC, ab6717, Abcam, San Francisco, CA, USA) for 1.5 h and dyed with Hoechst33342 (Beyotime biotechnology, Shanghai, China). The images were obtained with an Olympus IX71 microscope (Olympus) and color mergence was performed using ImageJ software (ImageJ version 1.44p, NIH, MD, USA).

### Immunohistochemical (IHC) staining

IHC staining was performed to determine the protein expression of p53, p21 and Ki67 in the xenograft tumor tissues and done as previously described 21.

### Animal studies

Four-week-old female BALB/c athymic mice were purchased from SLAC laboratory Animal Co., Ltd. (Shanghai, China) and randomly divided into four groups (6 mice per group). 6 × 10^6^ K1 cells or 3 × 10^6^ C643 cells were subcutaneously injected into the right groin of the mice to establish tumor xenografts. 7 days after injection, mice were treated with NVP-BEZ235 (25 mg/kg/day) and tumor size was measured every second day. Tumor volume was calculated using the equation: volume (mm^3^) = (length × width^2^)/2. The mice were then euthanized on day 15 post-injection and tumors were isolated. Tumor masses were weighed and then subjected to IHC analysis. All animal care and handling procedures were approved by the Animal Ethics Committee of Xi'an Jiaotong University.

### Statistical analysis

All results in the present study were presented as the mean ± standard deviation (SD). Statistical significance of difference between the results was assessed using a standard 2-tailed t test, conducted using SPSS statistical package (16.0, Chicago, IL, USA). A value of *p* < 0.05 was considered statistically significant.

## Results

### NVP-BEZ235 inhibits PI3K/Akt/mTOR signaling in human thyroid cancer cells

**Figure 1:**
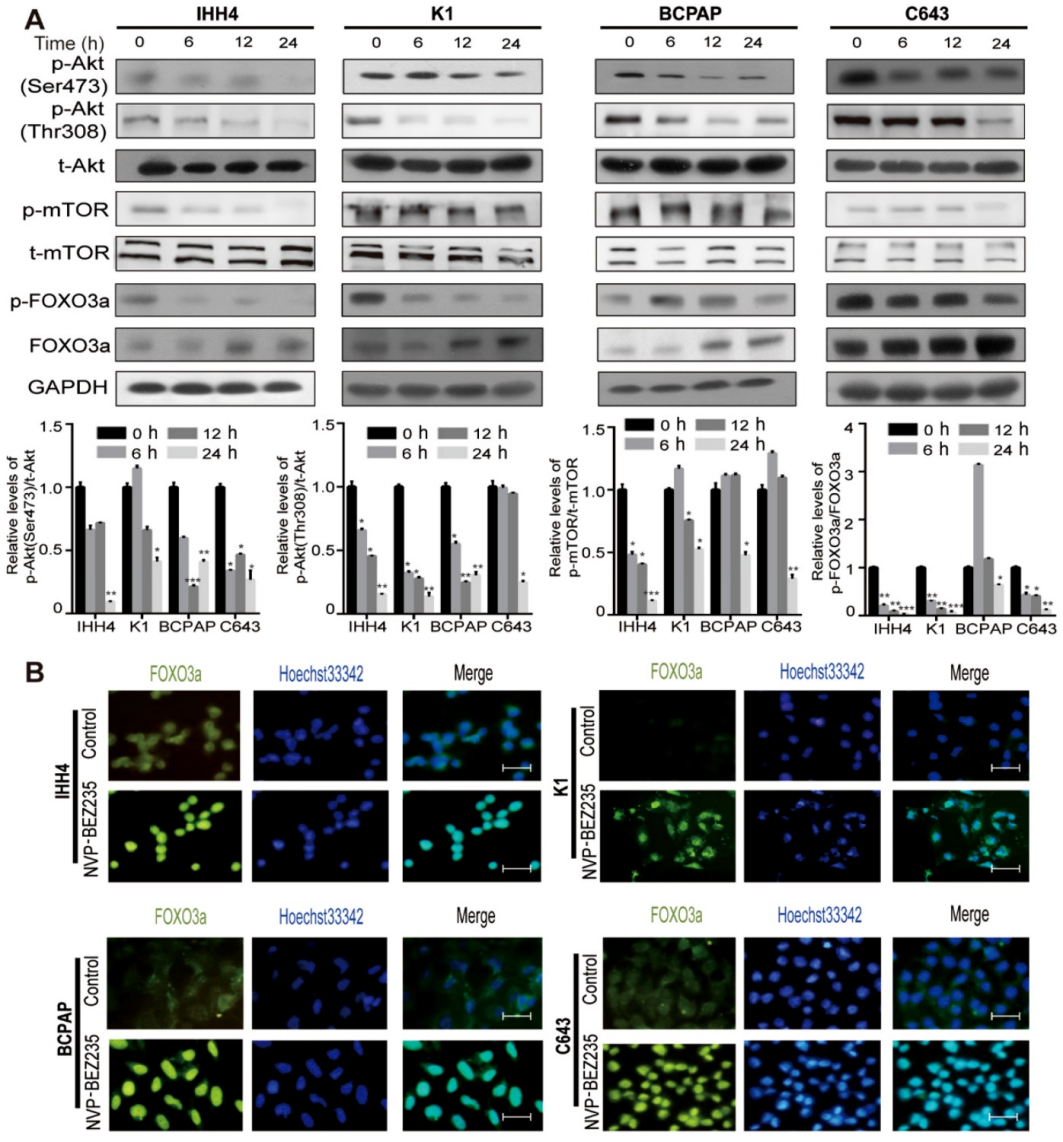
** The PI3K/Akt/mTOR signaling pathway is inhibited by NVP-BEZ235 in thyroid cancer cells. (**A) Western blot showed that NVP-BEZ235 treatment (200 nM) reduced phosphorylation of Akt (p-Akt; Ser473 and Thr308), mammalian target of rapamycin (p-mTOR) and Forkhead box O3 (p-FOXO3a) in IHH4, K1, BCPAP and C643 cells at 0 h, 6 h, 12 h and 24 h (upper). Relative protein levels were quantified by ImageJ software (lower). GAPDH was used as loading control. (B) Immunofluorescence showing that the increased nuclear translocation of transcription factor FOXO3a in IHH4, K1, BCPAP and C643 cells with NVP-BEZ235 treatment (200 nM) for 12 h. Scale bar = 20 μm. *p < 0.05, **p < 0.01, ***p < 0.001, respectively, NVP-BEZ235 versus control.

Excessive activation of the PI3K/Akt/mTOR signaling is frequently observed in thyroid tumorigenesis 22. Using western blot analysis, we first examined thyroid cancer cells response to NVP-BEZ235 by checking the PI3K/Akt/mTOR pathway activity. In both p53 wild type (IHH4 and K1) and mutant (BCPAP and C643) cell lines, 200 nM NVP-BEZ235 treatment decreased the phosphorylation of Akt (p-Akt; Ser473 and Thr308), mammalian target of rapamycin (p-mTOR) and Forkhead box O3 (p-FOXO3a), and increased the FOXO3a expression in a time-dependent manner (**Figure 1A**). Moreover, the results of immunofluorescence demonstrated that the nuclear translocation of transcription factor FOXO3a, a tumor suppressor and negative downstream effector of PI3K/Akt signaling 23, 24, was significantly increased after NVP-BEZ235 application (**Figure 1B**). These data indicated PI3K/Akt/mTOR signaling pathway was inhibited by NVP-BEZ235 treatment in both p53 wild type and mutant thyroid cancer cell lines.

### NVP-BEZ235 suppresses thyroid cancer cell proliferation

**Figure 2:**
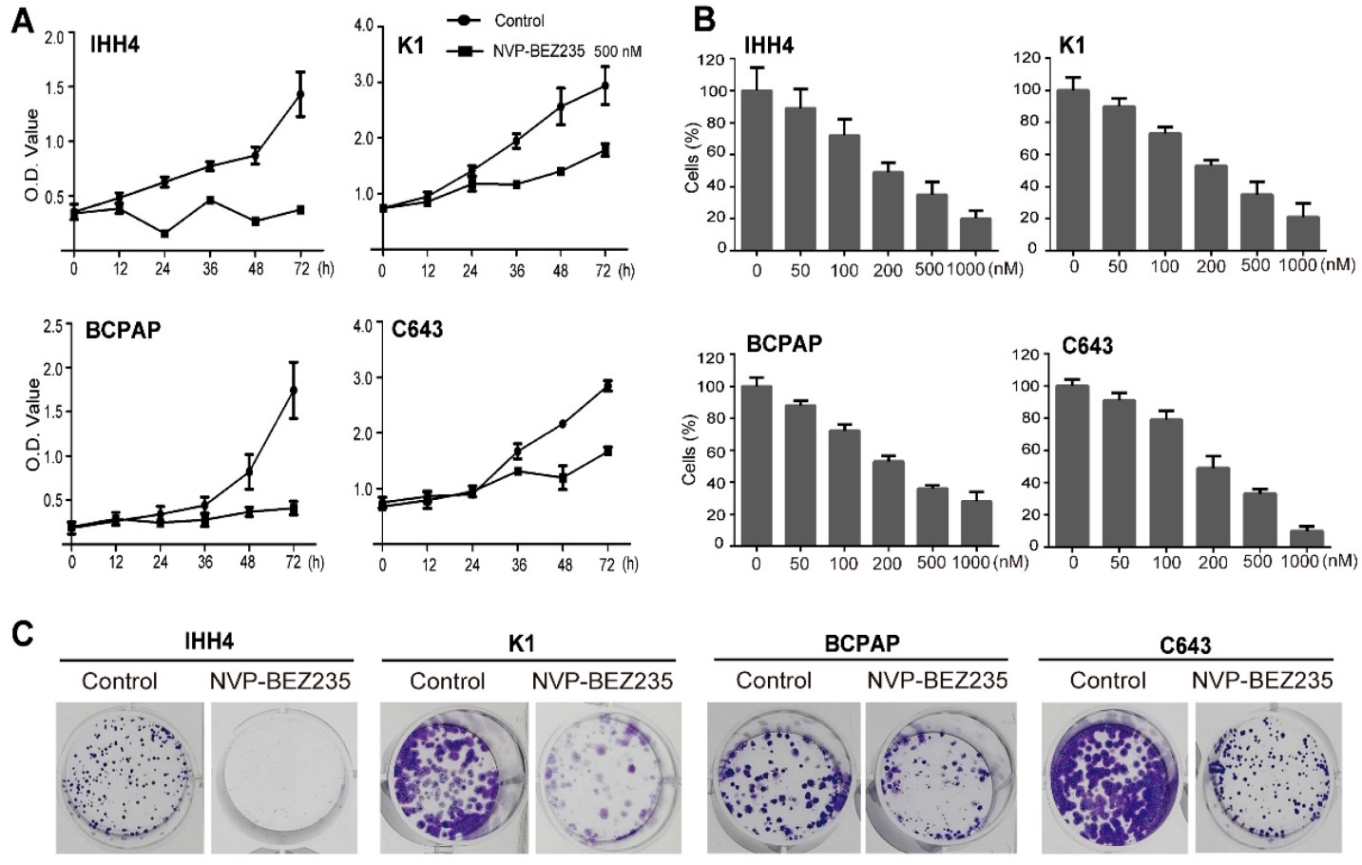
** NVP-BEZ 235 inhibits proliferation in a concentration- and time-dependent manner in thyroid cancer cells.** (A) MTT assays suggested that NVP-BEZ235 inhibited cell growth of IHH4, K1, BCPAP and C643 cells at 500 nM in a time-dependent manner. (B) Dose-response curves were drawn in IHH4, K1, BCPAP and C643 cells treated with NVP-BEZ235 from 50 nM to 1000 nM for 48 h. (C) Colony formation assay performed to assess proliferation of IHH4, K1, BCPAP and C643 cells. **p < 0.01, ***p < 0.001, NVP-BEZ235 versus control.

To evaluate the effect of NVP-BEZ235 on cell proliferation in thyroid cancer cells with different TP53 status, we cultured the cells with 500 nM NVP-BEZ235 or vehicle control, and using MTT assays to determine the cell viability on different time points (0, 12, 24, 36, 48 and 72 h). As shown in** Figure 2A**, NVP-BEZ235 inhibited cell proliferation in all the four thyroid cancer cell lines in a time-dependent manner. And gradient NVP-BEZ235 (0, 50, 100, 200, 500 and 1000 nM) application significantly slowed the growth of both p53 wild type and mutant thyroid cancer cells cultured for 48 h, showing a dose-dependent inhibitory effect (**Figure 2B**). The data in **Table 1** exhibited the corresponding IC_50_ of the four cell lines, indicating the half maximal inhibitory concentration of NVP-BEZ235 in IHH4, K1, BCPAP, and C643 were 38.9 nM, 70.3 nM, 123.5 nM, 221.0 nM, respectively. We got the phenomenon that the p53 wild type thyroid cancer cells were more sensitive to NVP-BEZ235 than p53 mutant thyroid cancer cells. In addition, as shown in **Figure 2C**, the long-term anti-proliferative activity on thyroid cancer cell proliferation was further confirmed by colony formation assay.

### NVP-BEZ235 treatment induces cell cycle arrest in human thyroid cancer cells

**Figure 3:**
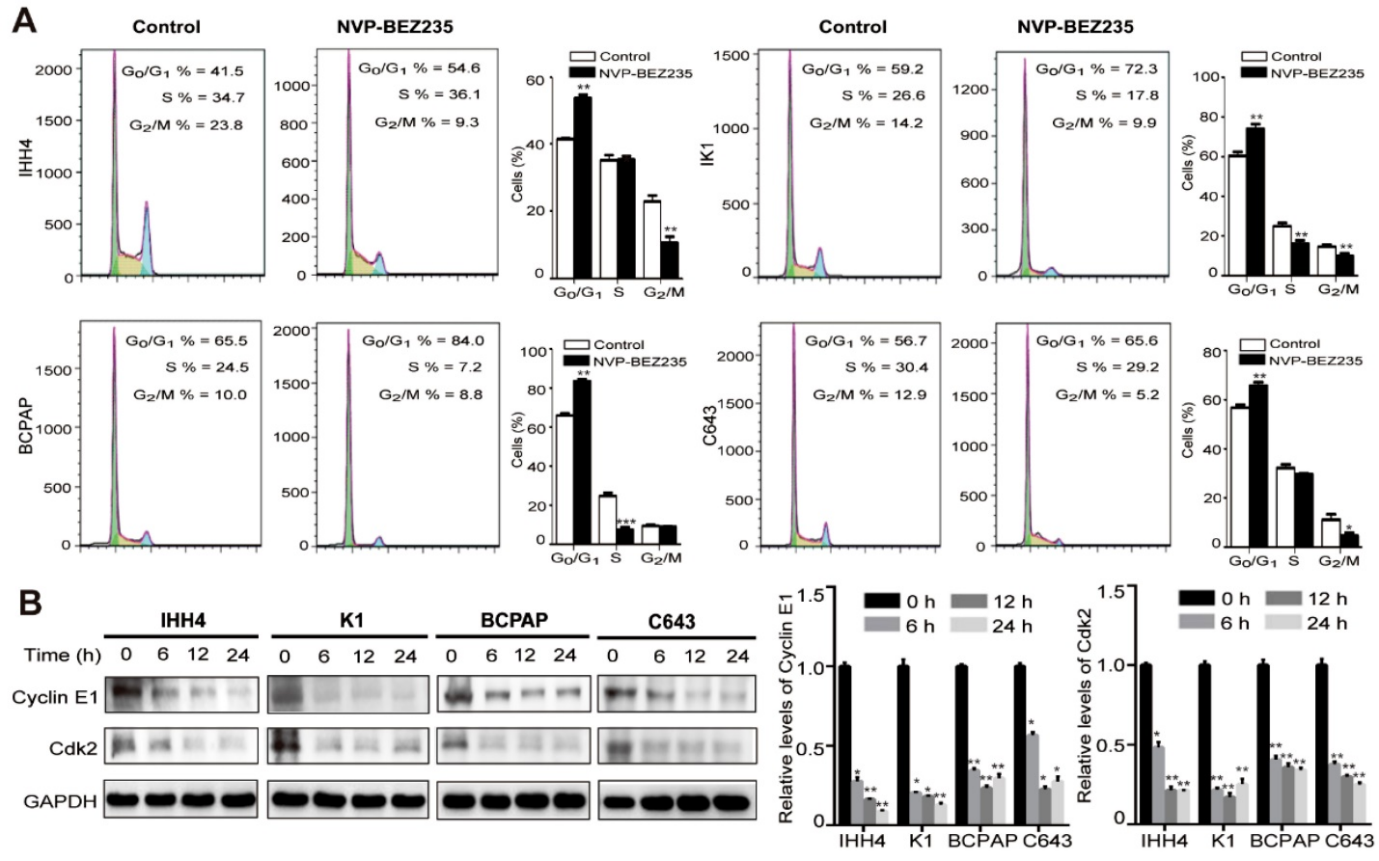
** NVP-BEZ235 induces cell cycle arrest in thyroid cancer cells.** (A) IHH4, K1, BCPAP and C643 cells were treated with 200 nM NVP-BEZ235 for 48 hours, respectively. DNA content was measured by flow cytometry to determine cell cycle fractions. The fraction of cells in each cell cycle phase was indicated in the figures. (B) The protein expressions of cyclin E1 and cyclin-dependent kinase 2 (Cdk2) were examined by western blot in IHH4, K1, BCPAP and C643 cells with 200 nM NVP-BEZ235 treatment (left). Relative protein levels were quantified by ImageJ software (right). *p < 0.05, **p < 0.01, ***p < 0.001, respectively, NVP-BEZ235 versus control.

Cell cycle -dependent mitotic rate is a key determinant of cell proliferation. Given the data above, we next tend to detect the cell cycle arrest after NVP-BEZ235 treatment. Compared with vehicle treated control cells, NVP-BEZ235 treatment significantly increased cell fractions at G_0_/G_1_ phase in all cell lines (**Figure 3A**). In specific, the G_0_/G_1_ phase proportion of IHH4 lifted from 41.5 % to 54.6 % (*p* < 0.01), from 59.2 % to 72.3 % (*p* < 0.01) for K1, from 65.5 % to 84.0 % (*p* < 0.001) for BCPAP, and from 56.7 % to 65.6 % (*p* < 0.01) for C643. Briefly, NVP-BEZ235 caused cell cycle arrest in the four thyroid cell lines regardless of *TP53* gene status, even more significant in cells carrying mutant *TP53*. In addition, cyclin E are known to interact with Cdk2 and are involved in triggering cell cycle activity in carcinogenesis 25. NVP-BEZ235 treatment significantly decreased the protein expression of cyclin E1 and Cdk2 in the human thyroid cancer cell lines in a time-dependent manner (**Figure 3B**).

### NVP-BEZ235 inhibits thyroid cancer cell growth through upregulation of p21

**Figure 4:**
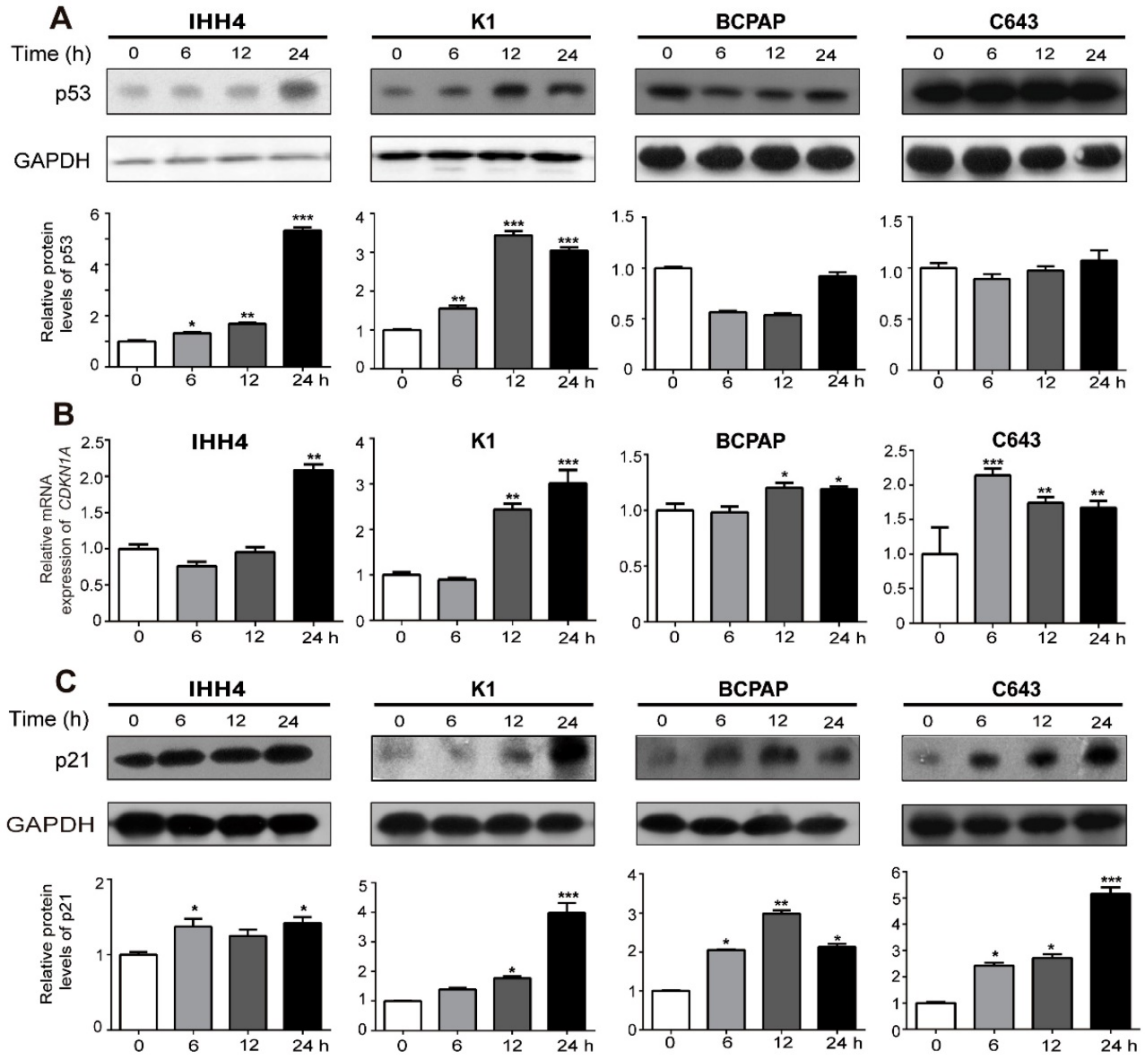
** NVP-BEZ235 up-regulates p21 in both p53 wild type and mutant thyroid cancer cells. (**A) p53 protein expression was determined by Western blot in IHH4, K1, BCPAP and C643 cells with 200 nM NVP-BEZ235 treatment. (B) *CDKN1A* mRNA expression was detected by qRT-PCR in IHH4, K1, BCPAP and C643 cells with 200 nM NVP-BEZ235 treatment. (C) p21 protein expression was tested by Western blot in IHH4, K1, BCPAP and C643 cells with 200 nM NVP-BEZ235 treatment. *β-actin* was used as a normalized control for qRT-PCR assay. GAPDH was used as loading control in western blot assays. *p < 0.05, **p < 0.01 and ***p < 0.001, respectively, NVP-BEZ235 versus control.

Cell cycle progression is a complex system involving cyclins, cyclin-dependent kinases, cyclin-dependent kinase inhibitors and orther associated proteins 26. P21, and p27 are both inhibitors of G_1_ cyclin-dependent kinases 27. And p21 is a transcription target of p53. Thus we tried to explore wether the inhibitory effect of cell growth induced by NVP-BEZ235 is up to p53, p21 or p27. As shown in western blot assays, NVP-BEZ235 treatment gradually increased the wild type p53 expression in IHH4 and K1 cells in a time dependent manner, while the mutant p53 expression was insusceptible, even slightly decreased in BCPAP and C643 (**Figure 4A,** representative results in the upper panel, and statistical analysis in the bottom). However, both *CDKN1A* (p21 coding gene) mRNA level and p21 protein expression levels were remarkably up-regulated by NVP-BEZ235 in all the four cell lines regardless of *TP53* status and independent of p53 expression levels (**Figure 4B and 4C,** for 4C, representative results in the upper panel, and statistical analysis in the bottom), suggesting the inhibitory effect of NVP-BEZ235 on thyroid cancer cell growth is related to transcriptional increase of p21. However, cells treated in the same condition showed no significant response of p27 to NVP-BEZ235 (**Supplementary Figure 1**).

**Figure 5:**
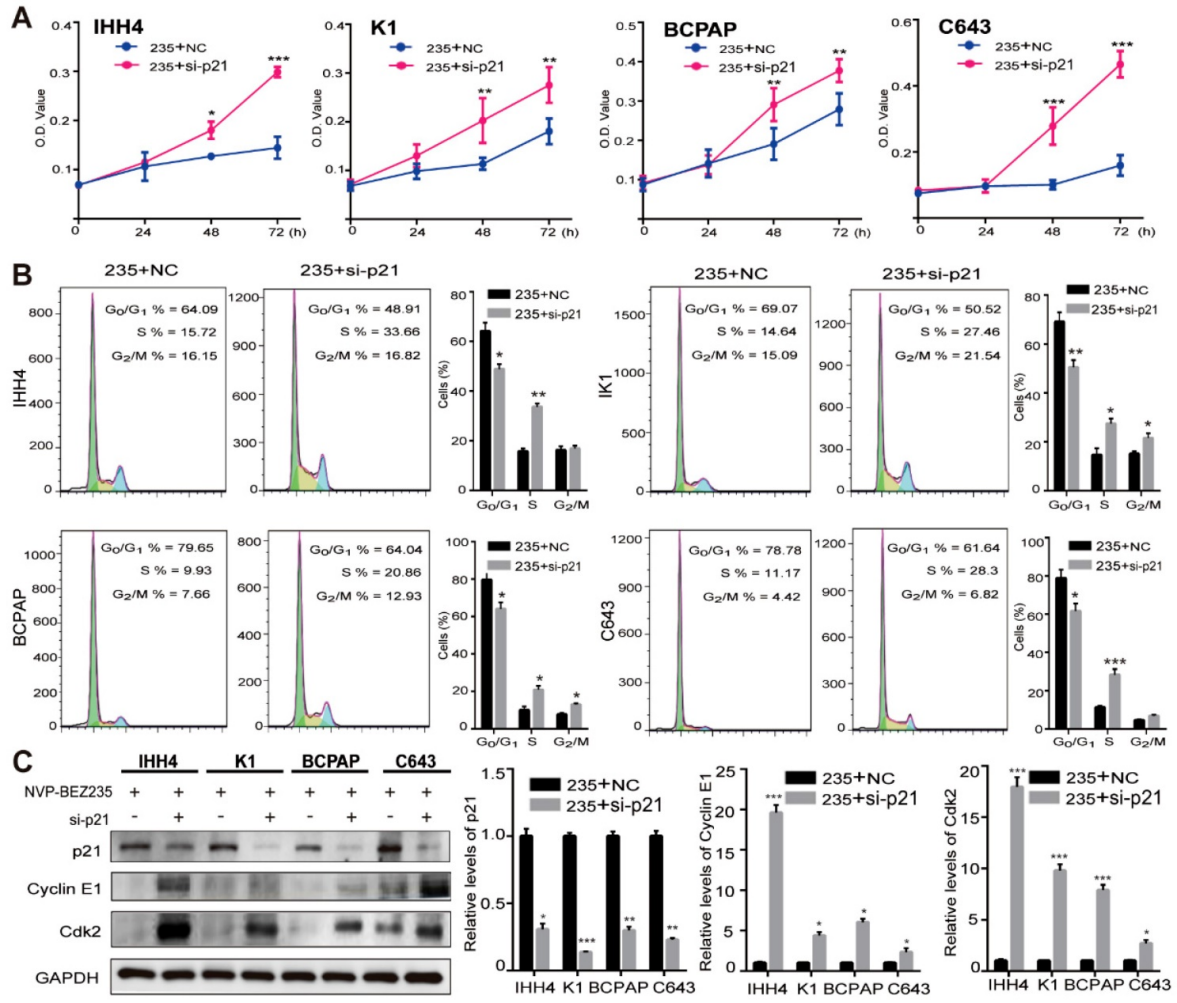
** p21 knockdown abolished the effect of NVP-BEZ235 in both p53 wild type and mutant thyroid cancer cells. (**A) MTT assays showed that the inhibitory effect of cell growth on IHH4, K1, BCPAP and C643 cells after NVP-BEZ235 treatment (200 nM) was abolished by p21 knockdown (si-p21). (B) Cell cycle arrest induced by NVP-BEZ235 (200 nM) was reversed by p21 knockdown (si-p21) in IHH4, K1, BCPAP and C643 cell lines. (C) p21 knockdown (si-p21) was performed in IHH4, K1, BCPAP and C643 cells and then cells were treated with NVP-BEZ235. The protein expressions of p21, cyclin E1 and Cdk2 were determined by Western blot (left). Relative protein levels were quantified by ImageJ software (right). GAPDH was used as loading control. 235, NVP-BEZ235; NC, Negative control. *p < 0.05, **p < 0.01, ***p < 0.001, respectively, 235 + si-p21 versus 235 + NC.

To further clarify the role of p21 in the cell growth inhibition induced by NVP-BEZ235, p21 knockdown was performed by siRNAs in the IHH4, K1, BCPAP and C643 cell lines (**Supplementary Figure 2**). Then the effect of p21 knockdown on cell proliferation after NVP-BEZ235 treatments was examined by MTT assays. As shown in** Figure 5A,** p21 knockdown significantly rescued the growth inhibitory effect of NVP-BEZ235 in all the four cell lines. Moreover, the results of flow cytometry suggested that p21 knockdown significantly attenuated cell cycle arrest induced by NVP-BEZ235 in all the four cell lines (**Figure 5B**). In specific, the G_0_/G_1_ phase proportion of IHH4 decreased from 64.09 % to 48.91 % (*p* < 0.01), from 69.07 % to 50.52 % (*p* < 0.01) for K1, from 79.65 % to 64.04 % (*p* < 0.01) for BCPAP, and from 78.78 % to 61.64 % (*p* < 0.01) for C643. Furthermore, p21 have been identified as a regulator capable of inhibiting cyclin E/Cdk2 complex 28. Accordingly, the suppression of cyclin E and Cdk2 was either reversed by p21 interfering, additionally demonstrated the cell growth inhibition induced by NVP-BEZ235 was through p21 function (**Figure 5C**). Therefore, p21 knockdown was capable to abolish the principal effect of NVP-BEZ235 on the thyroid cancer cells regardless of *TP53* status.

### GSK3β/β-catenin signaling inhibition contributed to p21 upregulation induced by NVP-BEZ235 in thyroid cancer cells

**Figure 6:**
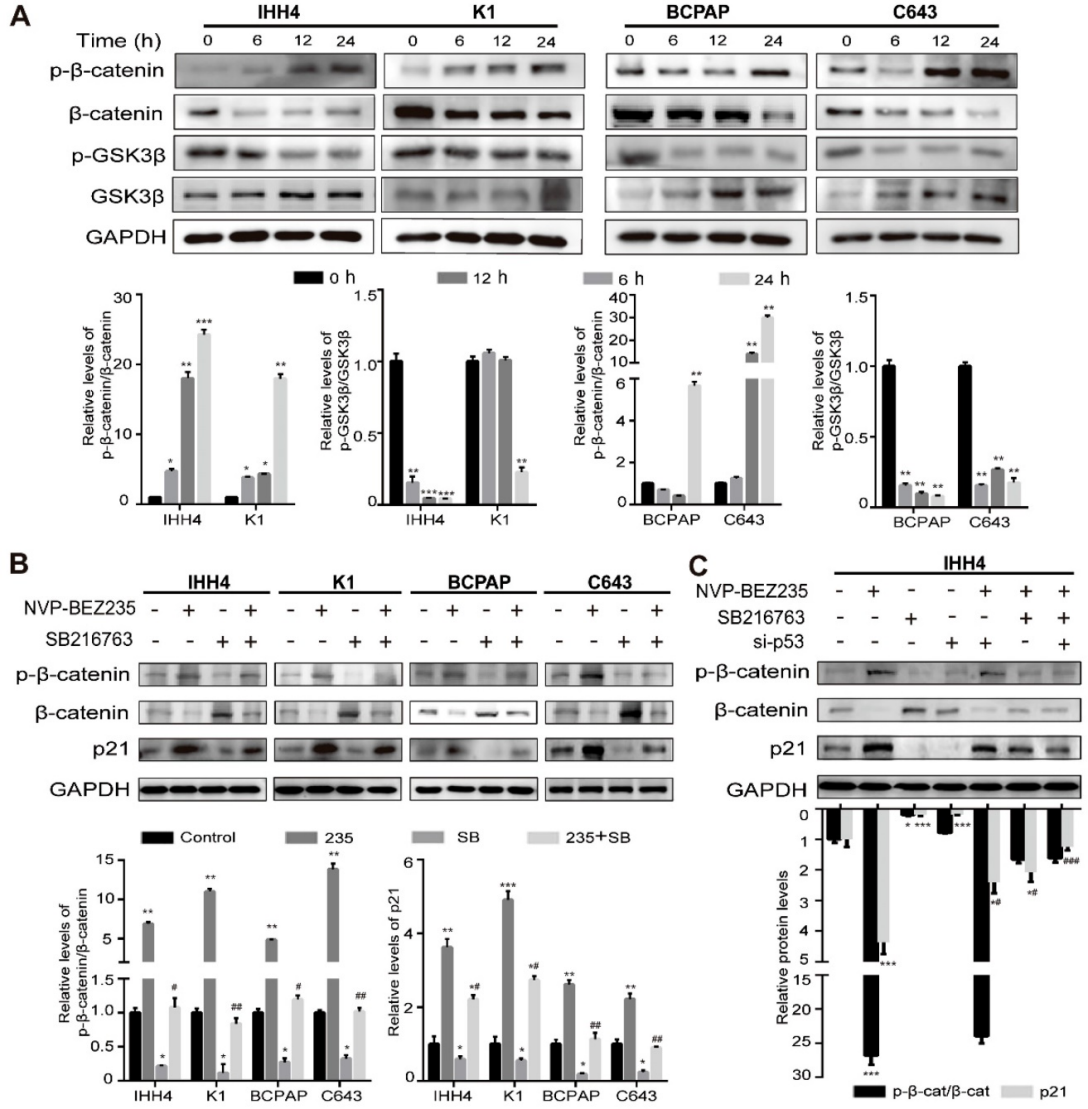
** NVP-BEZ235 inhibits GSK3β/β-catenin signaling and cell cycle proteins in human thyroid cancer cells.** (A) The protein expressions of phosphorylated β-catenin (p-β-catenin), β-catenin, phosphorylated glycogen synthase kinase 3 beta (p-GSK3β) and GSK3β were examined by Western blot in IHH4, K1, BCPAP and C643 cells with 200 nM NVP-BEZ235 treatment (upper). Relative protein levels were quantified by ImageJ software (lower). (B) The protein expressions of p-β-catenin, β-catenin and p21 were determined by Western blot in IHH4, K1, BCPAP and C643 cells treated with GSK3β inhibitor (10 μM; SB216763, SB) and NVP-BEZ235 (235) (upper). Relative protein levels were quantified by ImageJ software (lower). (C) The protein expressions of p-β-catenin, β-catenin and p21 were tested by Western blot in IHH4 cells with various treatments (upper). Relative protein levels were quantified by ImageJ software (lower). GAPDH was used as loading control. p-β-cat, p-β-catenin; β-cat, β-catenin. *p < 0.05, **p < 0.01, ***p < 0.001, ^#^*p* < 0.05 and ^##^*p* < 0.01, respectively. * refers to a group versus control, # refers to a group versus 235.

It is well known that p21 plays an essential role in anti-tumor progression in p53-dependent and -independent pathways 29, 30 and previous studies have shown that β-catenin was a negative regulator of p21 transcription 31, 32. As we found a p53-independent expression of p21 in NVP-BEZ235 treated* TP53* mutant cells, we supposed the glycogen synthase kinase β (GSK3β)/β-catenin signaling might be affected by NVP-BEZ235. The results of western blot analysis showed the phosphorylation of GSK3β was suppressed by NVP-BEZ235, accompanied by the kinase of GSK3β increased in all the four human thyroid cancer cell lines. And the target of GSK3 kinases, β-catenin was increasingly phosphorylated, suggesting its destabilization, and in accordance with the decrease of β-catenin amount (**Figure 6A**). These results clearly indicated that GSK3β/β-catenin pathway was inhibited by NVP-BEZ235 in both p53 wild type and mutant cells, and suggesting a potent mechanism of p21 upregulation. To identify the dependency of p21 expression on GSK3β/β-catenin pathway, IHH4, K1, BCPAP and C643 cells were treated with or without GSK3β inhibitor (SB216763) under the application of NVP-BEZ235. As shown in **Figure 6B**, GSK3β inhibitor abolished the principal effect of NVP-BEZ235 on p21 in p53 mutant cells, however partly prevented the increase of p21 induced by NVP-BEZ235 in p53 wild type cells. Furthermore, to determine the effect of p53 on p21 upregulation in p53 wild type cells, p53 knockdown by siRNAs was performed in IHH4 cells (**Supplementary Figure 3**). As shown in Figure 6C, western blot assays suggested that p53 knockdown or GSK3β inhibitor both partly prevented the p21 upregulation induced by NVP-BEZ235. However, the combination of p53 knockdown and GSK3β inhibitor completely reversed the increase of p21 induced by NVP-BEZ235 in IHH4 cells. These results indicated the inhibition of GSK3β/β-catenin played a key role in p53 mutant cells whereas p53 additionally contributes to the upregulation of p21 in p53 wild type cells.

### NVP-BEZ235 inhibits xenograft thyroid tumor growth in nude mice

**Figure 7:**
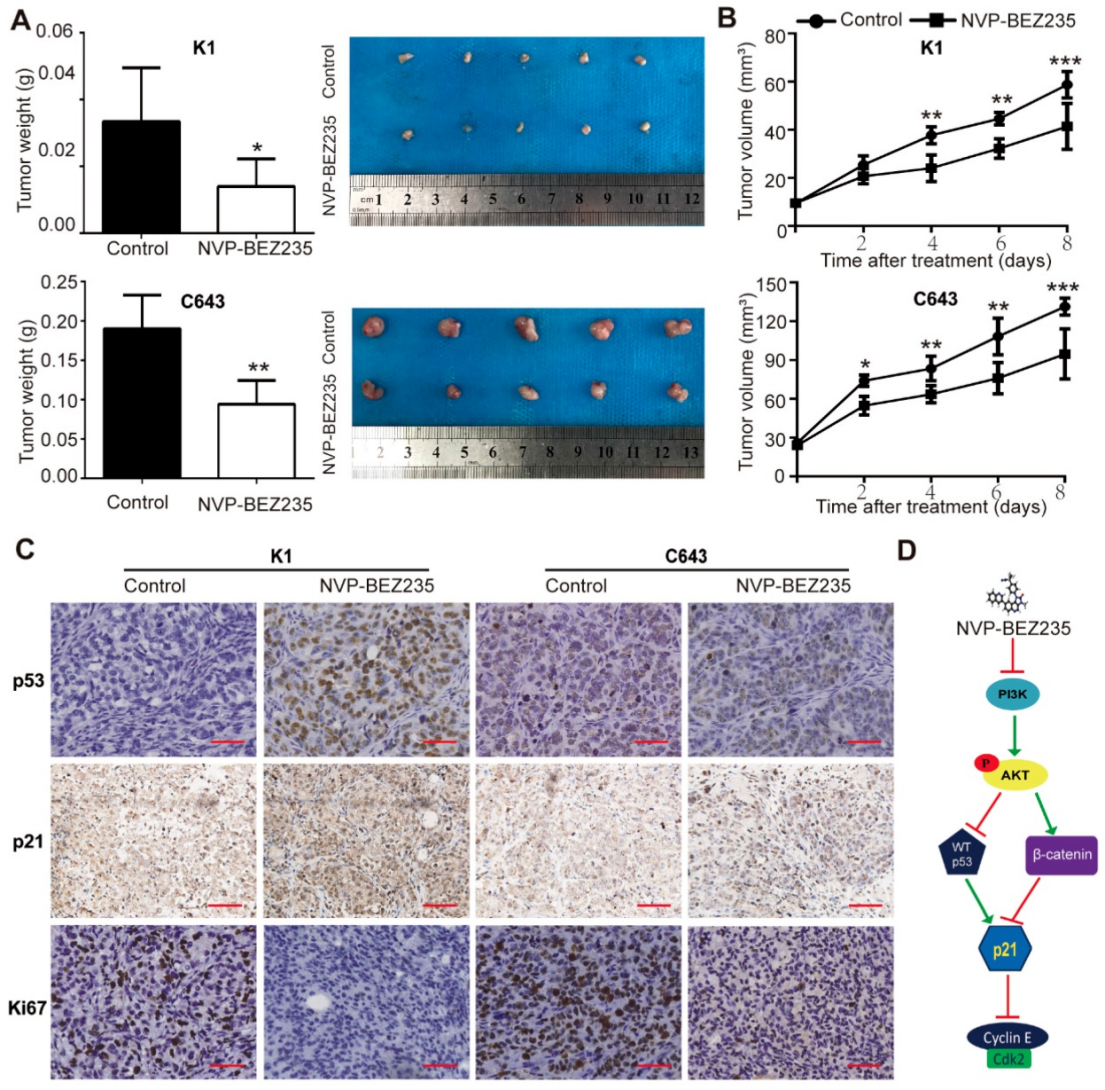
NVP-BEZ235 inhibits tumor growth in nude mice injected with K1 cells or C643 cells.** (**A) Tumor weight of mice with NVP-BEZ235 (25 mg/kg/day) treatment and the control group. (B) Tumor volume of mice with NVP-BEZ235 treatment and the control group. (C) IHC images of p53, p21 and Ki67 in xenograft tumors, scale bar = 20 μm. N = 5, *p < 0.05, **p < 0.01 and ***p < 0.001, respectively, NVP-BEZ235 versus control. (D) Schematic model of molecular mechanisms underlying tumor suppressive activity of NVP-BEZ235 in thyroid cancer. WT p53, wild type p53.

Finally, tumor xenografts were established to verify whether NVP-BEZ235 treatment inhibited tumor growth *in vivo*. Immuno-deficient mice were subcutaneously injected with p53 wild type K1 cells or p53 mutant C643 cells, treated with NVP-BEZ235 or vehicle, and the growth of the xenograft tumors were followed. Our data demonstrated both K1 and C643 tumors in mice given NVP-BEZ235 grew much slower than the control groups. Weight of the peeled tumors were also lower in the drug treated groups than the control groups (K1, 0.0334 ± 0.007222 g *vs.* 0.0140 ± 0.003674 g, N = 5,* p* < 0.05; C643, 0.1900 ± 0.01924 g *vs.* 0.0940 ± 0.01364 g, N = 5,* p* < 0.01) (**Figure 7A and 7B**).

Subsequently, the peeled tumors were subjected to paraffin embedded sections. The protein expressions of p53 and p21 in tumor sections were tested by IHC assays. In both K1 and C643 tumors, p21 expression was higher in NVP-BEZ235 treated mice compared with the controls, since an increased percent and stronger staining of p21 positive cells were observed. While only the mice bearing K1 tumors displayed greater portion and stronger staining of p53 positive cells, rather than C643 tumors. These results suggested a p53-independent mechanism of p21 up-regulation in NVP-BEZ235 treated C643 xenograft tumors, which was consistent with the results of *in-vitro* studies. Moreover, the mitotic cell marker, Ki67 was shown much weaker in the NVP-BEZ235 treated tumors than the control tumors, further suggested NVP-BEZ235 suppresses thyroid cancer cell proliferation (**Figure 7C**). Taken together, these results indicated that NVP-BEZ235 treatment inhibited tumor growth in mice injected with K1 cells or C643 cells and substantiate our findings that NVP-BEZ235 inhibits thyroid cancer growth independent of *TP53* status.

Altogether, the dual PI3K/mTOR inhibitor NVP-BEZ235 is efficacious against thyroid cancer, among which thyroid tumors with mutant p53 are critically dependent on the inhibition of GSK3β/β-catenin signaling, up-regulating p21 and then inhibiting cell growth in human thyroid cancer. However, p53 additionally contributes to the upregulation of p21 in p53 wild type cells (**Figure 7D**).

## Discussion

Tumorigenesis is a multistep process involving complicate molecular pathogenesis and mechanisms, including the genetic and epigenetic abnormities, involving tumor suppressor genes inactivation as well as oncogenes activation. Thus, great attention was given to molecular targeted cancer therapy in recent decades 8. The PI3K/mTOR/Akt signaling pathway has a fundamental role in numerous cancers of various histologic origin including thyroid cancer 5, 33, 34. This pathway appears abnormal to increase the expression and activity of Akt and its downstream effectors, disturbing many biological processes. NVP-BEZ235 as a novel treatment strategy, is a dual PI3K/mTOR inhibitor that has shown great efficacy in suppressing tumor growth in various cancers and currently in phase1/2 clinical trials 9. It is used alone or in combination to sensitize other drugs, as to achieve synthetic therapeutic effects with reduced side-effects. Yet, the molecular mechanisms that drive antineoplastic function are incompletely understood in thyroid cancer.

p53 have been identified as a tumor suppressor gene which integrates numerous signals controlling cell cycle arrest and apoptosis. The ability of p53 to eliminate excess, damaged or infected cells by apoptosis or induce cell cycle arrest, is critical for maintaining homeostasis of multicellular organisms 35-37. Nevertheless, loss or mutation of p53 occurs in approximately 50 % of all tumors 35, including thyroid cancer 6, 38. In our study, NVP-BEZ235 administration in thyroid cancer cell lines successfully inhibited Akt phosphorylation, hence, promoted downstream FOXO3a accumulation and nuclear translocation 24, and demonstrated remarkable anti-proliferation ability via inducing cell cycle arrest. However, we investigated the accumulation of p53 in wild type cell lines, but unaffected even slightly declined p53 was found in mutant cell lines. It is reasonable since Akt enhances MDM2 mediated proteasome degradation always fails for mutant p53 39. Meanwhile, p21, the primary effector of p53-mediated G_1_/S checkpoint control, was up-regulated, although differs in degree, in all the cell lines regardless of the volume change of p53 30. Accordantly, p21 expression was enhanced in the p53 wild type and mutant xenograft tumors in the NVP-BEZ235 treated mice. Additionally, previous research have disclosed that susceptibility to NVP-BEZ235 correlates with baseline expression of p27 16. However, we found that NVP-BEZ235 treatment failed to give rise to p27 response in IHH4, K1, BCPAP and C643 cells and this might be due to different cell lines used in our study. Taken together, we speculate that the antitumor effect in thyroid cancer cell lines harboring mutant *TP53* acts in a p53-independent manner.

p21 is known as a cyclin-dependent kinase (Cdk) inhibitor. It induces cell cycle arrest and represses the G1-to-S phase transition mainly by inhibiting the activity of the cyclin E/Cdk2 complex 27. The growth arrest mediated by p21 was reported to be negatively regulated by β-catenin via T-cell factor (TCF) or c-Myc transcriptional repression 31, 32, 40. In the present study, p21 was transcriptionally activated along with GSK3β/β-catenin pathway suppression when cells were treated with NVP-BEZ235. And this phenotype was observed in both p53 wild type and mutant thyroid cancer cells. Therefore, it's very potent to suppose a reasonable mechanism that NVP-BEZ235 induced G_0_/G_1_ cell cycle arrest depends on GSK3β/β-catenin/p21 cascade, though the contribution of wild type p53 to p21 in K1 and IHH4 cells is not eliminable. Indeed, the GSK3β inhibitor radically abolished the increase of p21 in mutant cells, and less effective in wild type cells in view of the activity of normal p53. Thus, we supposed the upregulation of p21 roots in GSK3β/β-catenin response and with/out the normal function of p53. However, the specific mechanisms how β-catenin regulated p21 needed to be further explored in our future studies. And p53 dependency analysis based on varying* TP53* status in the same cell line must help comprehend the NVP-BEZ235 efficacy.

In summary, the dual PI3K/mTOR inhibitor NVP-BEZ235 has a therapeutic effect on thyroid cancer, among which thyroid tumors with mutant p53 are critically dependent on the response of GSK3β/β-catenin signaling, up-regulating p21 and then inducing G_0_/G_1_ cell cycle arrest in human thyroid cancer.

## Supplementary Material

Supplementary figures.

## Figures and Tables

**Table 1:** The origins and genetic alterations of thyroid cancer cell lines

Cell lines	Origins	Genetic alternations			NVP-BEZ235 IC_50_ (nM )
		MAPK pathway	PI3K/Akt pathway	*TP53* status	
IHH4	PTC	*BRAF* (V600E)	Akt (E17K)	*TP53* (Wild-type)	38.9
K1	PTC	*BRAF* (V600E)	*PI3KCA* (E542K)	*TP53* (Wild-type)	70.3
BCPAP	PTC	*BRAF* (V600E)	*Akt1* copy gain	*TP53* (D259Y/K286E)	123.5
C643	ATC	*HRAS* (G13R)	--	*TP53* (R248Q/K286E)	221.0

## Abbreviations

PTC: papillary thyroid cancer
FTC: follicular thyroid cancer
WDTCs: well-differentiated carcinomas
PDTC: poorly differentiated thyroid cancer
ATC: anaplastic thyroid cancer
IHC: Immunohistochemical
mTOR: mammalian target of rapamycin
FOXO3a: Forkhead box O3
Cdk: cyclin-dependent kinase
TCF: T-cell factor
