# Supplementary material for: NVP-BEZ235 inhibits thyroid cancer growth by p53- dependent/independent p21 upregulation
Source: Int J Biol Sci. 2020 Jan 14;16(4):682–93. doi: 10.7150/ijbs.37592 (PMC6990918; doi:10.7150/ijbs.37592)
Supplement: Supplementary file 1 — Supplementary figures. [file ijbsv16p0682s1.pdf]

Supplementary Figure 1

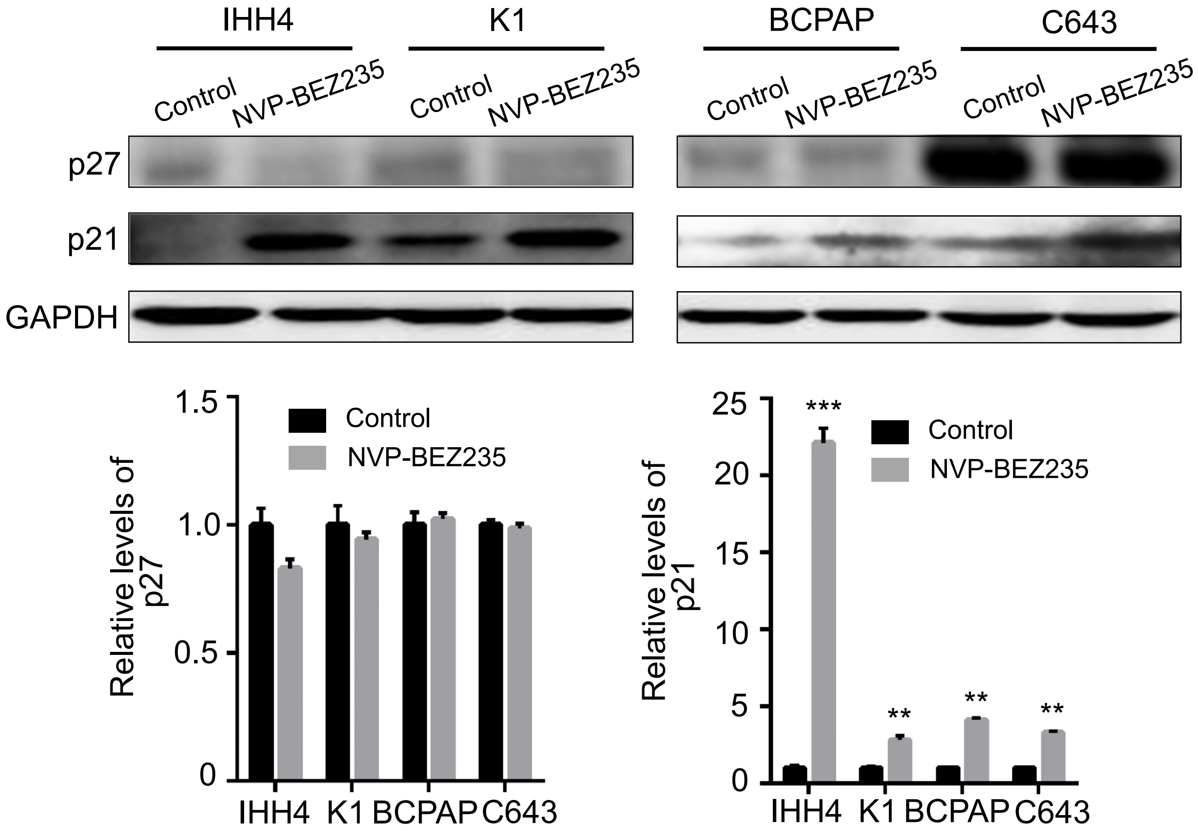

Supplementary Figure 1. Western blotting showed that NVP-BEZ235 treatment (200 nM) increased the expression of p21 but not influence p27 in IHH4, K1, BCPAP and C643 cells (upper). Relative protein levels were quantified by ImageJ software (lower). GAPDH was used as loading control

Supplementary Figure 2

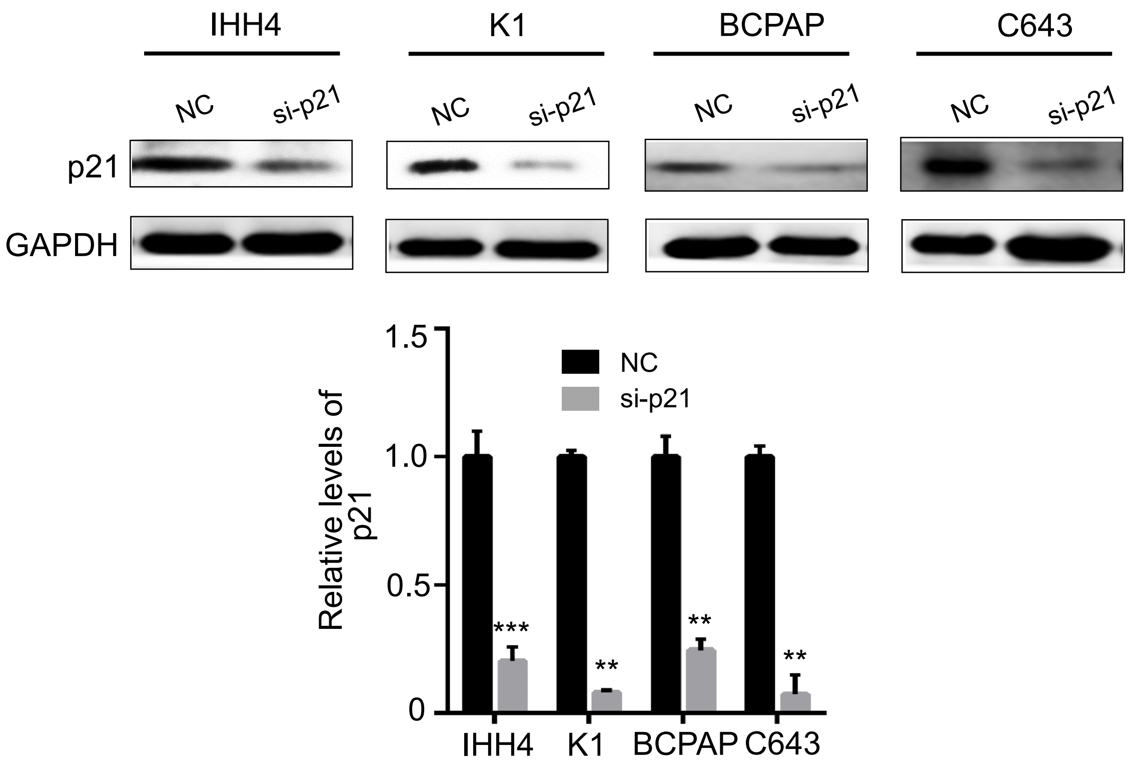

Supplementary Figure 2. Western blotting showed that the knockdown efficiency of p21 by siRNAs in IHH4, K1, BCPAP and C643 cells (upper). Relative levels of p21 were quantified by ImageJ software (lower). GAPDH was used as loading control.

Supplementary Figure 3

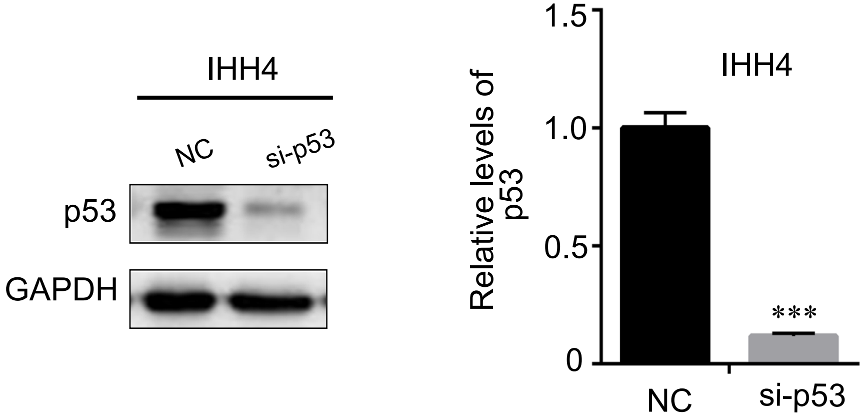

Supplementary Figure 3. Western blotting showed that the knockdown efficiency of p53 by siRNAs in IHH4 cells (left). Relative levels of p53 were quantified by ImageJ software (right). GAPDH was used as loading control.
